# Edward Grainger

**Published:** 1824-02

**Authors:** 


OBITUARY.—Lately deceased, EDWARD GRAINGER, Esq. Lecturer on Anatomy, &c.—We regret that the Communication made to us on this subject, was too late for us to avail ourselves of it.

